# Larval Migration Behaviour of *Busseola fusca* (Lepidoptera: Noctuidae) on Bt and Non-Bt Maize under Semi-Field and Field Conditions

**DOI:** 10.3390/insects11010016

**Published:** 2019-12-23

**Authors:** Andri Visser, Hannalene Du Plessis, Annemie Erasmus, Johnnie van den Berg

**Affiliations:** 1 Unit for Environmental Sciences and Management, IPM Program, North-West University, Potchefstroom 2520, South Africa; visseran3@gmail.com (A.V.); hannalene.duplessis@nwu.ac.za (H.D.P.); 2 Agricultural Research Council, Grain Crops, Private Bag X1251, Potchefstroom 2520, South Africa; erasmusA@arc.agric.za

**Keywords:** *Busseola fusca*, larval migration, Bt maize, insect resistance management, insect behaviour

## Abstract

*Busseola fusca* (Fuller) (Lepidoptera: Noctuidae) is a destructive pest of maize throughout the African continent. Bt maize is an effective control measure for this pest, however, selection pressure for resistance evolution is high. This necessitates the implementation of insect resistance management (IRM) strategies such as the high-dose/refuge strategy. This IRM strategy relies on the validity of several assumptions about the behaviour of pests during insect-hostplant interactions. In this study, the migration behaviour of *B. fusca* larvae was evaluated in a semi-field (greenhouse) and field setting. The effect of factors such as different Cry proteins, plant growth stage at infestation, and plant density on the rate and distance of larval migration were investigated over four and five week periods. Migration of the larvae were recorded by using both a leaf feeding damage rating scale and destructive sampling at the end of the trials. Results indicated that *B. fusca* larval migration success was significantly affected by plant growth stage and plant density—while limited larval migration was recorded in plots inoculated with larvae at a late growth stage (V10), higher plant density facilitated increased interplant migration. The results also suggest that *B. fusca* larvae do not migrate extensively (rarely further than two plants from the natal plant) and that larval mortality is high. Implications for IRM strategies are discussed.

## 1. Introduction

The African stem borer, *Busseola fusca* (Fuller) (Lepidoptera: Noctuidae) is a destructive pest of maize and sorghum throughout sub-Saharan African [1,2]. Multiple applications of broad-spectrum insecticides have historically been the norm for the control of stem borers in commercial maize farming systems in South Africa until the commercial release of genetically engineered Bt maize in 1998 [3,4]. Since then, Bt maize cultivars have provided effective control of *Chilo partellus* (Swinhoe) (Lepidoptera: Pyralidae), *Sesamia calamistis* (Hampson) (Lepidoptera: Noctuidae), and *B. fusca* [5,6]. 

The predominant threat to the sustainability of Bt maize technology is the evolution of resistance by pest populations [7,8,9,10]. The widespread use and efficacy of Bt crops result in high and continuous selection pressure for resistance evolution throughout the growing season [11,12]. During the first decade of commercially available Bt crops (1996–2005), only three cases of field-evolved insect resistance to Cry proteins were reported. By 2016, this number has increased to 16 species with resistance to a range of Cry proteins both in single-gene and pyramid events [13]. *Busseola fusca* was one of the first maize pests to exhibit field-evolved resistance to Cry 1Ab maize and did so within a mere eight years after the release of Bt maize in South Africa [14]. However, Tabashnik and Carrière [13] also reported on 17 cases in which no decrease in susceptibility of target pests to Bt crops were observed. In the majority of these cases, susceptibility levels remained unchanged for over a decade. It is therefore possible to deploy Bt crops sustainably, if an effective insect resistance management (IRM) strategy is in place. 

While there are several IRM strategies, the high-dose/refuge (HDR) strategy is the most widely applied [15]. This strategy requires the expression of Bt toxins in a dose high enough to kill heterozygous-resistant individuals, and a source of non-Bt host plants (refuge area) in close proximity to the Bt field, which acts as a source of homozygous susceptible individuals [16]. Effectively, only rare homozygous resistant individuals of the target pest survive on the Bt crop, and these individuals most likely mate with the abundant homozygous susceptible individuals that originate from the refuge area. The heterozygous offspring of these mating pairs are then eliminated by the high-dose of Bt toxins, thereby reducing the chance that the resistance genes are passed on to the next generation [13]. 

A non-Bt refuge area can be structured in different ways in relation to the Bt crop field. A refuge can be planted as a separate block, as strips within the Bt field or as a perimeter surrounding the Bt field [13]. These are referred to as structured refuges. More recently, producers have also been offered the option of using unstructured refuges, also known as seed mixtures/blends, refuge-in-a-bag, or mosaic refuges [17]. The latter implies mixing of non-Bt and Bt seed at a certain ratio within the bag which results in the non-Bt seeds being planted randomly within a crop field [17,18].

The HDR-strategy is a generic protocol that is based on several assumptions regarding pest–plant interactions, as well as the interactions between resistant and susceptible individuals in the pest population. To improve the efficacy of the HDR-strategy, the design of the refuge (type of refuge, structure, distance from Bt crop, etc.) must take into account the biology and behaviour of the specific target pest [19]. It is widely accepted that seed mixtures will not be an appropriate strategy to use with a pest that is highly mobile during the larval stages, since this could lead to larvae being exposed to sub-lethal doses of Bt toxins, accelerating resistance evolution [18,20,21,22]. The behavioural traits of *B. fusca* that are especially relevant to refuge design are the preferences of moths for ovipositional hosts, preference of larvae for the particular host plant, and subsequent larval migration behaviour. 

Generally, insect pest species have a narrow host range and display strong taxonomic conservatism in their choice of host plants [23]. These host plant preferences are based on the chemical characteristics (e.g., nutrient content, primary, and secondary metabolites), as well as plant physical characteristics (e.g., spines, trichomes). These plant attributes are used by foraging insects to assess the suitability of a plant and leads to the development of preferences for host plants by insect species [23,24]. *Busseola fusca* moths and larvae possess sensory structures able to detect plant compounds that influence host plant choice [25,26]. It is therefore possible that the presence of Bt-toxins in maize leaf tissue can be detected by foraging larvae and that this may affect their feeding and migration behaviour. Such effects of the presence of Cry proteins in Bt crops have been demonstrated for several pest species including the following noctuids: *Helicoverpa zea* (Boddie), *Heliothis virescens* (Fabricius), and *Spodoptera frugiperda* (J.E. Smith) [27]. When a host plant is not preferred by larvae, foraging and migration within and between plants is likely to continue [28]. 

Larval migration generally takes two forms: crawling or ballooning [23]. Ballooning, also referred to as silking, is when larvae produce silk strings to dangle themselves from plant structures until they come into contact with a different plant/plant structure or are carried off by the wind [18,19]. As larvae mature, ballooning becomes increasingly difficult due to their size and weight. Later stage larvae therefore disperse mostly by crawling [28]. Larval density, host plant suitability and environmental factors are all factors that could influence the rate and success of larval migration [19,28].

Since agriculture in Africa is practiced largely on small holder and subsistence farming plots, a generic IRM strategy developed for industrial agriculture will most likely not be effective, due to the challenges provided by implementing structured refuges [29,30,31,32]. Instead, it is essential that IRM approaches are tailored to best fit the behaviour of the target pest species and the scale of production [33]. In order to develop practical and appropriate IRM strategies, this study was conducted to generate information on the migration behaviour of *B. fusca* larvae and to determine which factors may impact their migration and survival under field conditions. 

## 2. Materials and Methods 

### 2.1. Stock Colonies of B. fusca

Two Cry1Ab-resistant populations, collected in the Vaalharts region in 2016 (VAA16-R) and Harrismith region (HAR18-R), were used in this study. The HAR18-R population was used in the field experiment, whereas the greenhouse experiment was conducted using the VAA16-R population. 

Diapausing larvae of a Cry1Ab-resistant population (VAA16-R) of *B. fusca* were collected from maize stubble in the Vaalharts area (S 27°51′8.55″, E 24°48′20.059″) during August of 2016. The larvae were placed in 15 ℓ plastic containers (37 cm (L) × 29 cm (W) × 19 cm (H)), with 50 larvae per container. The containers were kept in a temperature-controlled room at 24 ± 2 °C, ambient humidity and a 14L:10D photoperiod. Sheets of paper were placed inside the containers to provide shelter for the larvae. The contents of each container were sprayed generously with distilled water daily to terminate diapause and initiate pupation, following the methods described by Van Rensburg and Van Rensburg [34].

Larvae of the HAR18-R population were collected from a maize field (growth stages V6–V10) in the Harrismith area (S 28°12′54″; E 29°4′51.3″) during January 2018. These larvae were kept in similar containers and environmental conditions as described above. Larvae were reared to the pupal stage on non-Bt maize stems. 

A Cry1Ab-susceptible population (EC18-S) of diapausing *B. fusca* larvae were collected from maize stubble in a non-Bt field (S 33°4′28.153″; E 27°38′41.204″) during late August 2018 in the Eastern Cape province, where *B. fusca* larvae are reported to be highly susceptible to Cry1Ab maize [35,36]. The same process described above was followed to store and terminate larval diapause of the EC18-S population.

Since *B. fusca* populations in different maize cultivation regions of South Africa are in contact with each other, continuous gene-flow occur between the populations [37]. Therefore, it is safe to assume that the reaction of the larvae in each of the trails will be similar in *B. fusca* populations in all geographic areas and are not singular phenomenon specific to a population of a specific geographic area.

### 2.2. Production of Neonate B. fusca Larvae for Experiments

Calatayud et al. [25] found that *B. fusca* moths that have been reared for several generations in a laboratory showed reduced ability to discriminate between host- and non-host plants. To control for this effect in the current migration assays, only moths originating from the field-collected larvae and their F_1_ larval progeny were used in this study. 

*Busseola fusca* egg batches were obtained by rearing field-collected populations according to the methods described in [27]. The egg batches were placed in 50 mL plastic containers with mesh-infused lids, which was then kept in a glass desiccator (30 cm diameter) with the RH maintained at 70 ± 5% by means of a potassium hydroxide solution [38]. The desiccator was kept in a rearing chamber (26 °C ± 1 °C, RH 65 ± 5%, and a photoperiod of 14L:10D) and was checked daily for hatching neonate larvae.

The methods used to confirm the status of resistance/susceptibility of the VAA16-R and HAR18-R populations are described in Visser et al. [27]. The *B. fusca* population in the Vaalharts area is known to be resistant to Cry1Ab maize [36,39,40].

### 2.3. Maize Plants

Three maize hybrids representing two different Bt treatments and a control were used in this study. These were a single-gene Bt hybrid expressing Cry1Ab protein (DKC 8012B; MON810), as pyramid hybrid expressing Cry1.105 + Cry2Ab2 proteins (DKC 8012 B GEN; MON89034), and a near-iso non-Bt hybrid (DKC 8010; non-Bt)).

### 2.4. Experiment Protocols

#### 2.4.1. Greenhouse Experiment

The aim of this experiment was to determine the effect of the presence of Bt-toxin and plant growth stage on the migration behaviour of Cry1Ab-resistant *B. fusca* larvae (both within maize plants as well as between maize plants) under semi-field conditions. 

This experiment was conducted in a ventilated, irrigated greenhouse (38 m × 19.5 m) at the Agricultural Research Council—Grain Crops Institute (ARC—GCI) in Potchefstroom, South Africa. Two temperature loggers (Model DS1921G iButton, ColdChain Thermodynamics, Sandton, South Africa) were used to monitor the ambient temperature and humidity which were recorded as 26 °C and 65% respectively for the duration of the experiment. The ambient photoperiod during the study was approximately 12L:12D. Seed were planted directly into the soil and watering was done as needed. Each of the three hybrids was allocated eight plots (24 plots in total). Each plot (1.5 × 6 m, 1.0 to 1.5 m spacing between plots) consisted of three rows (0.75 m inter- and 0.20 m intra-row spacing), with a non-Bt maize plant as the centre plant in the centre row (irrespective of hybrid). This non-Bt maize plant was marked and would later serve as the inoculated plant. 

Of the eight plots planted with each hybrid, four were allocated to the early infestation treatment, and the other four to the late infestation treatment. The 24 plots were arranged according to a randomized complete block design. 

Inoculation was done at either the V4-leaf stage (to simulate an early infestation) or V10 leaf stage (to simulate a late infestation, one week prior to tasselling). Growth stages are determined by the number of visible leaf collars (thus 4 visible leaf collars at V4 stage). The V4-leaf stage was reached 2–3 weeks after seedling emergence (plant height approximately 50 cm), and V10-leaf stage was reached 5 weeks after seedling emergence (plant height approximately 170 cm). The non-Bt maize plant in the centre of each plot was inoculated with 30 neonate larvae of the Cry1Ab-resistant VAA16-R population. Neonate larvae were placed directly into the whorls of the maize plants. Directly preceding inoculation of the centre plant in each plot, QuickStix immunochromatographic GE strip tests (EnviroLogix, Portland, ME, USA) was used to verify that the plants designated for inoculation were non-Bt plants.

The severity of larval feeding damage to all plants in each plot was assessed at weekly intervals for five weeks following inoculation. The 1–9 Davis rating scale was used as basis for this rating [41]. Late-infested plants were also assessed for damage for three consecutive weeks after inoculation. During each damage assessment, the number of plants exhibiting whorl damage symptoms was recorded, as well as the position of the plant within the plot and the damage rating for each plant. This was done to determine the spread of plants exhibiting leaf damage symptoms in plots over time as a proxy of the migration of the larvae. This method has previously been used successfully by Ndjomatchoua et al. [42]. 

At the last assessment, all maize plants were cut open to determine the number of larvae present in each plant. The length of tunnels caused by borer larvae in each stem was determined and the position (internode number) of this damage recorded for each stem. 

#### 2.4.2. Field Experiment

The aim of this experiment was to determine the effect of Bt-toxin and plant density on the migration behaviour of Cry1Ab-resistant *B. fusca* larvae under field conditions. The field experiment was conducted in an open, irrigated field (1 ha) at the ARC—GCI. This experiment had four treatments, each replicated four times: non-Bt and MON810 maize hybrid treatments, each planted at a high and low plant density. The high plant density plots consisted of nine rows (0.75 m inter- and 0.18 m intra-row spacing), while the low plant density plots had 7 rows (1.2 m inter-and 0.42 m intra-row spacing). 

The high plant density treatment represented the density of a typical maize field under irrigation (80,000 plants/ha), whereas the low plant density plots represented a typical planting density in dryland maize fields (20,000 plants/ha) in South Africa [43]. Plots were spaced 1.50 m apart. Similar to the greenhouse experiment, a non-Bt maize plant was the centre plant of the centre row (irrespective of maize hybrid), which would later serve as the inoculated plant. The plots were arranged in a randomized complete block design and were watered as required. Four rows of non-Bt maize were planted as a border around the trial.

Every plant in each plot was inspected for damage symptoms caused by natural infestation prior to the start of the experiment. The border rows of non-Bt maize that surrounded the field experiment were inspected for natural stem borer infestation at weekly intervals throughout the trial, since natural infestation in the treatment plots could complicate the interpretation of the damage rating results. The non-Bt maize plants in the middle of each plot were inoculated with 30 neonate larvae of the Cry1Ab-resistant *B. fusca* population four weeks after seedling emergence. Directly preceding inoculation of these plants, GE strip tests were used to verify that the plants designated for inoculation were non-Bt plants. 

The number of damaged plants per plot as well as their position in each plot was recorded at weekly intervals for four weeks following inoculation. This was done to determine the spread of damage symptoms in plots as a proxy of the migration of the larvae. The damage assessments were done on a 1–9 scale as described above. At the last assessment, plants displaying stem-borer damage were cut open to determine the number of larvae present in each plant. 

Although no natural infestation of stem borers was observed in the plots prior to the inoculation with *B. fusca* larvae, a number of plants in the border rows did show symptoms of stem borer damage by the second week after the main plots were inoculated with *B. fusca* larvae. These turned out to be as a result of *Chilo partellus* (Swinhoe) (Lepidoptera: Crambidae) larval damage. At the same time, leaf feeding damage started to appear on plants at the edges of the non-Bt plots. It was decided to let the trial continue and the position and rating of all damaged plants were noted meticulously. During the destructive sampling it was found that the plants that were infested by *C. partellus* all occurred in the outer 2 to 3 rows of the non-Bt plots and that it never included those infested with *B. fusca* larvae in the central areas of the treatment plots. The plants that were damaged by *C. partellus* could therefore easily be identified and separated from the plants damaged by *B. fusca* larvae and were therefore not included in the data analyses for this experiment.

### 2.5. Data Analysis

**Figure 1:**
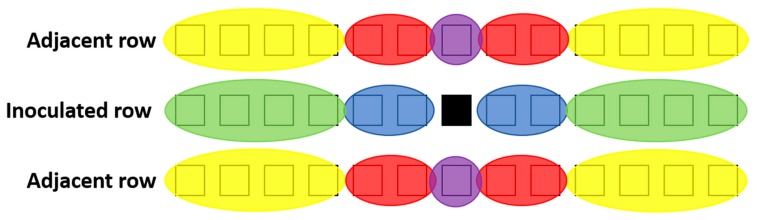
Pooling of plants for distance intervals. Each square represents a maize plant, with the centre row of squares representing the inoculated row and the adjacent rows located on either side thereof. In the inoculated row, Distance 0 includes only the inoculated plant itself (black square). Distance 1 includes the next two plants in both directions from the inoculated plant (encircled in blue). Distance 2 includes the furthest 4 plants on either end of the row (encircled in green). In the two adjacent rows, Distance 0 includes only the two plants directly adjacent to the inoculated plant (encircled in purple). Distance 1 includes the next two plants in both directions (encircled in red). Distance 2 includes the last 4 plants on either end of the adjacent rows (encircled in yellow).

All data were analysed using Statistica v13.3 (TIBCO Software Inc. 2017, Palo Alto, CA, USA). In both the greenhouse and the field experiment, no feeding damage were recorded, nor were any larvae recovered, further than 6 plants away from the initial inoculated plant. In the field experiment with multiple plot rows, feeding damage and larvae were recorded only within the inoculated row and the two immediate adjacent rows. Therefore, the analyses in these two experiments were limited to a total of 39 plants in each plot (the inoculated plant and the 38 plants in its immediate surrounds) (Figure 1).

#### 2.5.1. Weekly Monitoring of Plants for Leaf Damage: Damage Frequency

The number and position of damaged plants in each plot was recorded weekly for the duration of the experiment. First, the total number of damaged plants were analysed by means of a factorial ANOVA, with the following four main factors: plant density (high and low—only in the field experiment analysis), hybrid (non-Bt and MON810, with the addition of MON89034 in the greenhouse experiment analysis), week (W1–W5 and W1–W4 after inoculation in the greenhouse and field experiment analyses, respectively) and row (inoculated and adjacent). A Bonferroni post-hoc tests was used to identify significant differences between means.

Next, the number of damaged plants were pooled into three distance intervals relative to the inoculated plant (Distance 0, 1 and 2), as illustrated in Figure 1. The percentage of damaged plants within each distance interval was then calculated and compared between treatments using a General Linear Model (GLM)-analysis. The same factors that were used in the ANOVA were included in the GLM-analysis, with the addition of ‘distance’ as a factor. The GLM-analysis did not use a full factorial analysis, but only included the main factor of distance (Distance 0–2), with the interaction of distance with each of the other factors, as well as the interaction of distance with all possible combinations of these factors. A Bonferroni post-hoc tests was used to identify significant differences between means.

#### 2.5.2. Weekly Monitoring of Plants for Leaf Damage: Damage Rating

The average damage rating (1–9 scale) was calculated using only the damaged plants per plot. Damage ratings were analysed by means of a factorial GLM-analysis with the following five factors: plant density (high and low—only in the field experiment analysis), hybrid (non-Bt and MON810, with the addition of MON89 in the greenhouse experiment analysis), week (W1–W5 and W1–W4 in the greenhouse and field experiment analyses, respectively), row (inoculated and adjacent), and distance (Distance 0–2). A Bonferroni post-hoc test was used to identify significant differences between means. 

For the greenhouse experiment, the damage frequency and damage severity rating analyses were conducted separately for the early and late infestation datasets. This was because the late infestation dataset only coincided with the last three weeks of the early infestation dataset.

#### 2.5.3. Destructive Sampling: Larval Survival and Migration Distance

At the end of both the experiments, the number of surviving larvae in each stem was recorded. The data were analysed by means of a factorial GLM-analysis, incorporating the following five factors: plant density (high and low—only in the field experiment analysis), hybrid (non-Bt and MON810, with the addition of MON89034 in the greenhouse experiment analysis), week (W1–W5 and W1–W4 in the greenhouse and field experiment analyses, respectively), row (inoculated and adjacent), and distance (Distance 0–2). A Bonferroni post-hoc tests was used to identify significant differences between means.

## 3. Results

### 3.1. Greenhouse Trial: Effect of Plant Growth Stage

#### 3.1.1. Weekly Monitoring of Damage Symptoms: Damage Frequency

**Figure 2:**
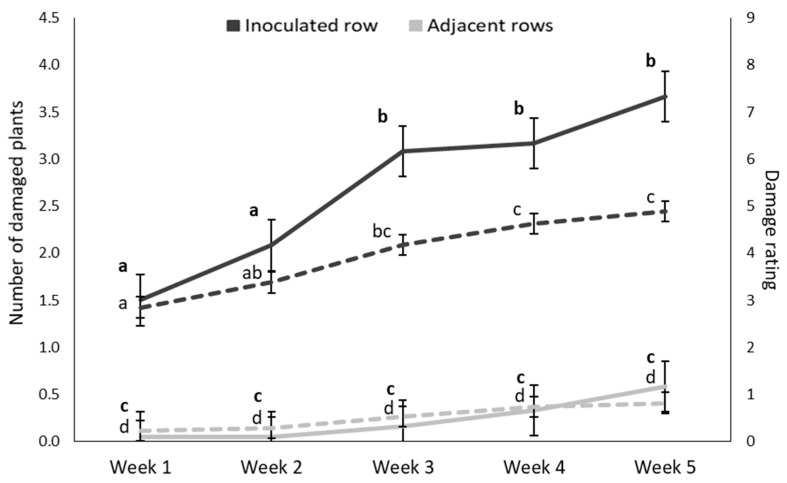
Mean (±SE) number of damaged plants (solid lines) and damage rating of plants (dashed lines) over time in maize plots in the glasshouse trial after the inoculation of the centermost plant in each plot (12 plots) with 30 MON810-resistant *B. fusca* larvae (VAA16-R) at V4-growth stage. Means marked with different letters are significantly different (*p* < 0.05).

The results of the ANOVA and GLM statistical analyses of the plant damage frequency data for infestation at both the early and late growth stages are provided in Table S1. The ANOVA results of the number of plants per plot that exhibited whorl damage in the early infested plots indicated that both the main effects (week and row) and the interaction effect between row and week was significant (*p* < 0.05). This result is illustrated in Figure 2, which shows that the mean number of damaged plants was significantly higher in the inoculated row compared to the adjacent rows throughout the five-week period. The number of damaged plants increased from weeks two to three, after which it levelled off. The average number of damaged plants in the rows adjacent to the inoculated plant remained very low over the five-week period (Figure 2).

**Figure 3:**
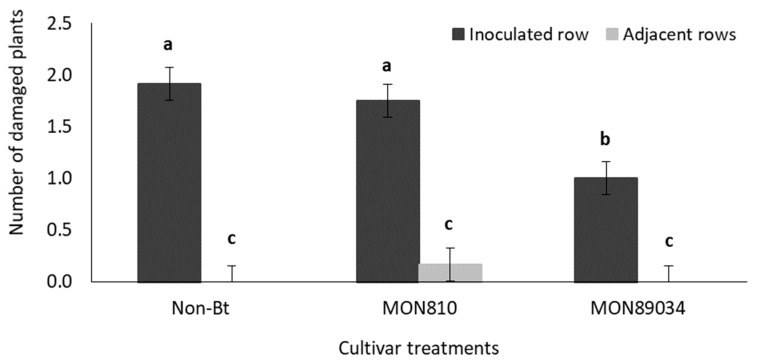
Mean (±SE) number of damaged plants in the inoculated and adjacent rows of non-Bt (DKC 8010), MON810 (DKC 8012 B), and MON89034 (DKC 8012 B GEN) maize treatment plots in the late infestation of glasshouse trial. Bars marked with different letters are significantly different (*p* < 0.05). The centermost maize plant in each treatment plot was inoculated with 30 MON810-resistant *B. fusca* larvae (VAA16-R) at V10-growth stage and damage frequency was recorded weekly for a three-week period. Each hybrid treatment was replicated four times.

For the late infestation, the main effects (hybrid and row) as well as the interaction effect was significant (*p* < 0.05) (Table S1). The average number of damaged plants in the adjacent rows were inconsequential when compared to that of the inoculated row for all three hybrid treatments (Figure 3). Significantly more plants in the inoculated row of the non-Bt and MON810 plots were damaged compared to that of the inoculated row in the MON89034 plots (Figure 3). 

To determine the distance that larvae migrated during the trial, the number of damaged plants were pooled into three distance (plant number) intervals relative to the inoculated plant (Plant 0, Plant 1–2, Plant 3–6), as described in Figure 1. The GLM-analyses indicated that the interaction between the main factors (row and distance) were statistically significant for both early and late inoculated plants (Table S1). 

**Figure 4:**
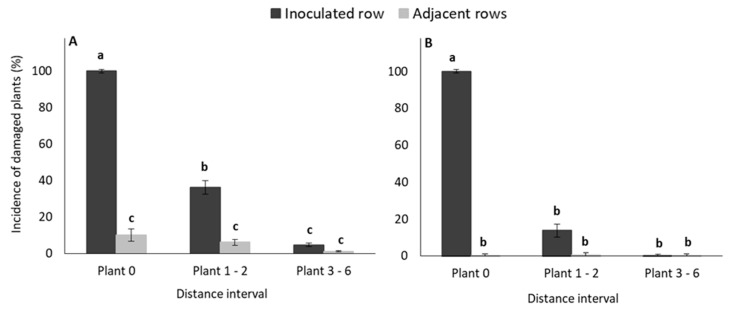
Mean (± SE) percentage of damaged plants recorded in each distance interval (Figure 1) in (**A**) early infestation maize treatment, and (**B**) late infestation maize treatment plots in the glasshouse trial. Bars marked with different letters are significantly different (*p* < 0.05). The centermost maize plant (Plant 0) in each treatment plot was inoculated with 30 MON810-resistant neonate *B. fusca* larvae (VAA16-R) at V4-growth stage and damage frequency was recoded weekly for a five-week (early infestation) and three-week (late infestation) period.

In plots where plants were inoculated during the early vegetative growth stage, the incidence of damaged plants in the inoculated row decreased significantly as the distance from the inoculated plant increased. The incidences of damaged plants in the three distance intervals in adjacent rows were very low and did not differ (Figure 4). Figure 4, which reflects results of late inoculated plants, indicates that only the inoculated plants (Plant 0, inoculated row) exhibited any symptoms of larval feeding damage.

#### 3.1.2. Weekly Monitoring of Damage Symptoms: Damage Rating

Analysis of the early infestation data indicated that all the main effects significantly (*p* < 0.05) influenced the severity of larval damage to whorl leaves (Table S1). Significant *p*-values were also observed for the two-way interactions between week and row, hybrid and distance, week and distance, row and distance, and the three-way interaction between hybrid, row, and distance.

**Figure 5:**
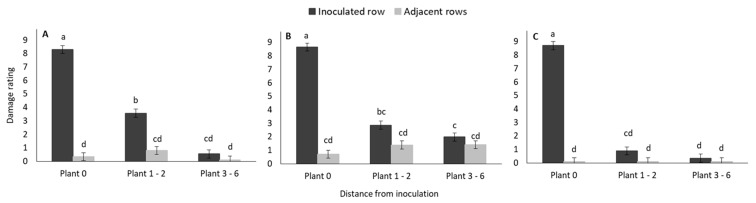
Mean (±SE) damage rating of early infested plants in three distance intervals (Figure 1) in the inoculated and adjacent rows of (**A**) non-Bt (DKC 8010), (**B**) MON810 (DKC 8012 B), and (**C**) MON89034 (DKC 8012 B GEN) maize treatment plots in the glasshouse trial. Bars marked with different letters are significantly different (*p* < 0.05). The centremost maize plant in each treatment plot was inoculated with 30 neonate MON810-resistant *B. fusca* larvae (VAA16-R) at V10-growth stage and damage rating was recoded weekly for a three-week period. Each hybrid treatment was replicated four times.

The mean damage rating of inoculated plants (Inoculated row, Plant 0) in early infested plots did not differ between the three maize hybrid treatments (Figure 5). The damage ratings of plants in the Plant 1–2 interval of the inoculated row were significantly higher for the non-Bt (Figure 5A) hybrid than for the MON89034 hybrid (Figure 5C). For the furthest interval of plants (Plants 3–6) in the inoculated row, the mean damage rating was significantly higher for MON810 plants than MON89034 plants. However, the leaf damage rating for plants of the non-Bt hybrid at the furthest distance interval did not differ significantly from that of plants of either of the two Bt hybrids. The mean damage rating of plants in the adjacent rows of all three hybrid treatments were similar and lower than that in the inoculated row (Figure 5). 

The gradual increase in damage rating over time, of plants in the early-inoculated plots is shown in Figure 2. The mean damage rating in the adjacent row were consistently lower than in the inoculated row and did not increase markedly over time. 

The results of the GLM-analysis for average damage rating of late inoculated plants are provided in Table S1. All the main effects were significant (*p* < 0.05), with the exception of hybrid. Significant *p*-values were also observed for the three-way interactions between week, row, and distance, and several two-way interactions, including the interaction between hybrid and distance, week and row, week and distance, and row and distance. 

**Figure 6:**
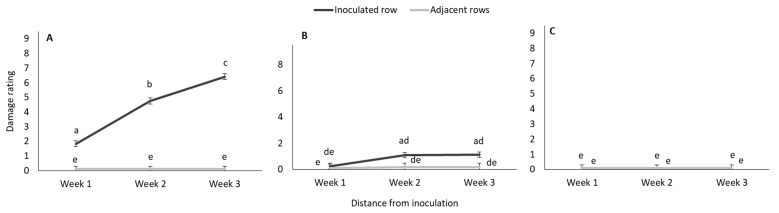
Mean (±SE) damage rating of late infested plants at three distance intervals (Figure 1) (**A**) Plant 0, (**B**) Plants 1-2, and (**C**) Plants 3–6 in the inoculated and adjacent rows of maize treatment plots in the glasshouse trial. Means marked with different letters are significantly different (*p* < 0.05). The centermost maize plant in each treatment plot was inoculated with 30 neonate MON810-resistant *B. fusca* larvae (VAA16-R) at V10-growth stage and damage frequency was recoded weekly for a three-week period. Each hybrid treatment was replicated four times.

While the mean damage rating of plants that were inoculated during the late vegetative stage increased significantly over time (inoculated row, plant 0) (Figure 6A) this was not the case for damage to other plants (Figure 6B,C). 

**Figure 7:**
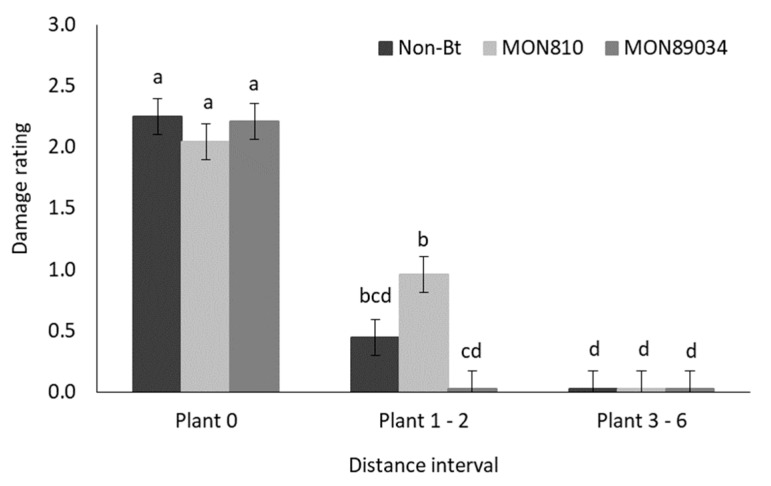
Mean (±SE) damage rating of late infested plants at three distance intervals from the inoculated plant (Figure 1) in the non-Bt (DKC 8010), MON810 (DKC 8012 B), and MON89034 (DKC 8012 B GEN) maize treatment plots in the glasshouse trial. Bars marked with different letters are significantly different (*p* < 0.05). The centermost maize plant in each treatment plot was inoculated with 30 neonate MON810-resistant *B. fusca* larvae (VAA16-R) at V10-growth stage and damage frequency was recoded weekly for a three-week period. Each hybrid treatment was replicated four times.

The mean damage rating of the non-Bt inoculated plants in the late infestation plots were similar for all three maize hybrid treatments (plant 0) (Figure 7). However, the damage rating of MON810 plants were significantly higher than that of MON89034 plants at the plant 1–2 interval. No damage was recorded on any plants in the plant 3–6 interval for any of the hybrids.

#### 3.1.3. Destructive Sampling: Larval Survival

Results of the GLM-analysis of larval survival in early and late infestation treatment plots are provided in Table S2. The main effects (row and distance) as well as the interactions were significant (*p* < 0.05). 

**Figure 8:**
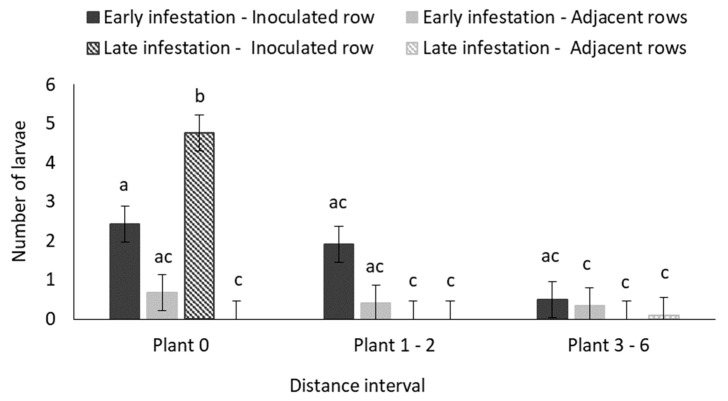
Mean (±SE) number of larvae of early and late infested plants in three distance intervals (Figure 1) in the inoculated and adjacent rows of maize treatment plots in the glasshouse trial. Bars marked with different letters are significantly different (*p* < 0.05). The centremost maize plant in each treatment plot was inoculated with 30 neonate MON810-resistant *B. fusca* larvae (VAA16-R) at V4- and V10-growth stage, and the number of surviving larvae in each plant were recorded by destructive sampling of the treatment plots at the end of the trial. Each hybrid treatment was replicated four times for both the early and late infestation treatments.

The highest number of surviving larvae was recovered from the inoculated non-Bt plants (Figure 8) in the centre of the maize treatment plots. Very few larvae were recovered at the second distance interval (plants 1–2) and the furthest distance interval (plants 3–6) in both the inoculated and adjacent rows in all treatments.

### 3.2. Field Trial: Effect of Plant Density

#### 3.2.1. Weekly Monitoring of Damage Symptoms: Damage Frequency

The ANOVA results of the incidence of plants with larval feeding damage symptoms are provided in Table S3. All main effects were significant (*p* < 0.05), and the mean number of damaged plants were significantly higher in the high-density treatment plots ($\bar{x}$ = 2.9 ± SE 0.21) than in the low-density treatment plots ($\bar{x}$ = 1.94 ± SE 0.21). The mean number of damaged plants were also significantly higher in the non-Bt hybrid ($\bar{x}$ = 2.96 ± SE 0.21) than the MON810 hybrid plots ($\bar{x}$ = 1.87 ± SE 0.21), and higher in the inoculated row ($\bar{x}$ = 3.48 ± SE 0.21) than in adjacent rows ($\bar{x}$ = 1.35 SE = 0.21). Further analysis of the main effect “Week” indicated that the mean number of damaged plants were significantly higher in Week 3 ($\bar{x}$ = 3.06, SE = 0.26) than in Week 1 ($\bar{x}$ = 1.9 ± SE 0.26).

**Figure 9:**
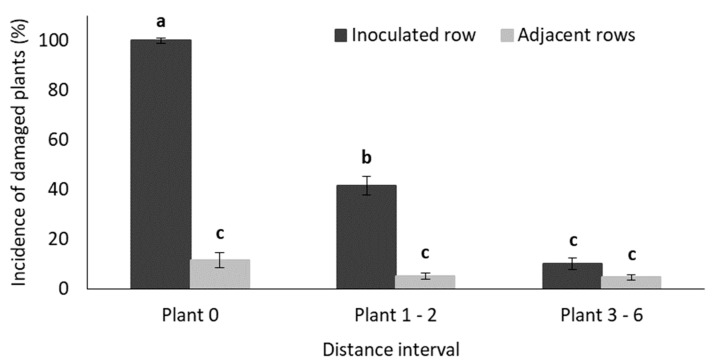
Mean (±SE) percentage of damaged plants recorded in each distance interval (Figure 1) in treatment plots in the field trial. Bars marked with different letters are significantly different (*p* < 0.05). The centremost maize plant (Plant 0) in each treatment plot was inoculated with 30 MON810-resistant neonate *B. fusca* larvae (HAR18-R) at V4-growth stage and damage frequency was recoded weekly for a three-week period. The data table indicate the actual number of plants in each distance interval, with the mean number of plants damaged in each interval.

Table S3 shows results of the GLM analyses which aimed to determine the distance that larvae migrated during the trial, as described Figure 1. The main factor of distance as well as the interaction between row and distance were statistically significant (*p* < 0.05). In the row that contained the inoculated plant, the incidence of damaged plants decreased as the distance from the inoculated plant increased and it differed significantly between distance intervals (Figure 9). The three distance intervals in the adjacent rows all exhibited a low incidence of damaged plants (Figure 9).

#### 3.2.2. Weekly Monitoring of Damage Symptoms: Damage Rating

Results of the GLM-analysis for average damage rating indicates that all main effects were significant (*p* < 0.05) (Table S3). *p*-values of the significant main effects as well as two-way and three-way interactions are provided in Table S3. 

**Figure 10:**
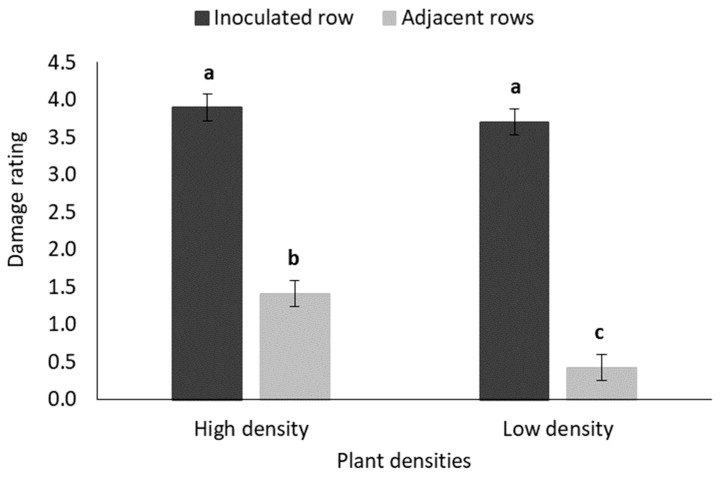
Mean (±SE) damage rating of maize plants in the inoculated and adjacent rows of high-density (0.75 m inter-row; 0.18 maize intra-row spacing) and low-density (1.2 m inter-row; 0.42 m intra-row spacing) treatment plots in the field trial. Bars marked with different letters are significantly different (*p* < 0.05). The centremost maize plant in each treatment plot was inoculated with 30 MON810resistant neonate *B. fusca* larvae (HAR18-R) at V4-growth stage and damage rating was recoded weekly for a three-week period. Each density treatment was replicated eight times.

The mean damage rating was significantly higher in inoculated rows than in the adjacent rows of both the high- and low-density treatment plots (Figure 10). Although the mean damage rating did not differ between the inoculated rows of the two plant density treatments, the mean damage rating was significantly lower in the adjacent rows of the low-density plots than in the high-density plots (Figure 10). 

**Figure 11:**
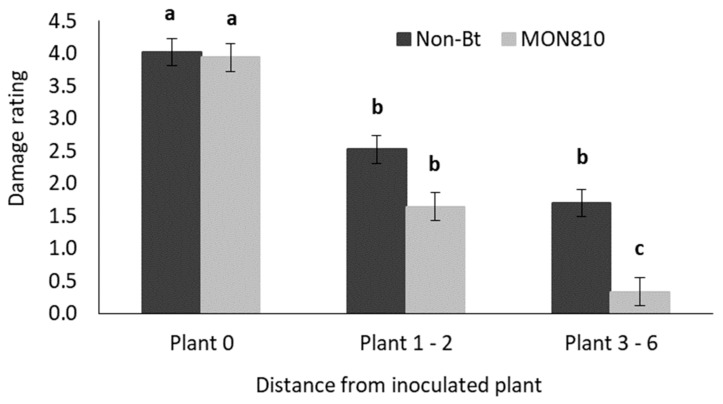
Mean (±SE) damage rating of maize plants at three distance intervals (Figure 1) in non-Bt (DKC 8010) and MON810 (DKC 8012 B) treatment plots in the field trial. Bars marked with different letters are significantly different (*p* < 0.05). The centermost maize plant in each treatment plot was inoculated with 30 MON810-resistant neonate *B. fusca* larvae (HAR18-R) at V4-growth stage and damage rating was recoded weekly for a three-week period. Each hybrid treatment was replicated eight times.

Figure 11 shows that the mean damage rating decreased as the distance from the inoculated plant increased in both the non-Bt and the MON810 hybrid plots. The mean damage ratings for both the non-Bt and MON810 hybrids did not differ significantly at the first two distance intervals (plant 0 and plants 1–2). However, MON810 plants at the furthest distance interval (plants 3–6) suffered significantly lower damage compared to plants at a similar distance in the non-Bt maize plots. 

**Figure 12:**
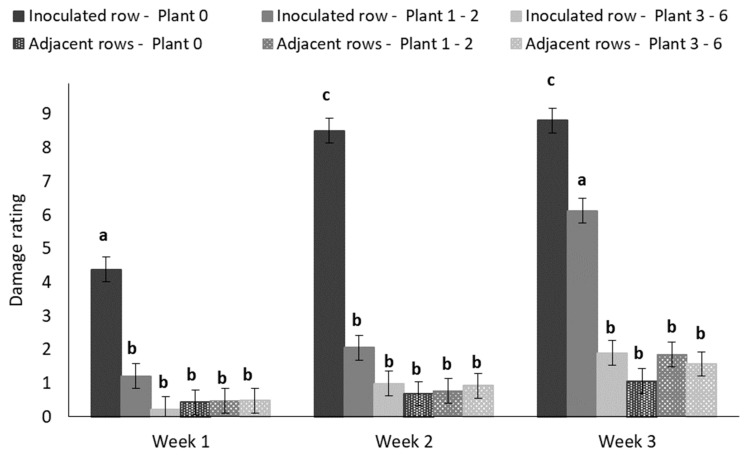
Mean (±SE) damage rating of maize plants over a 3-week period at three distance intervals (Figure 1) in the inoculated and adjacent rows of treatment plots in the field trial. Bars marked with different letters are significantly different (*p* < 0.05). The centermost maize plant in each treatment plot was inoculated with 30 MON810-resistant neonate *B. fusca* larvae (HAR18-R) at the V4-growth stage and damage was rated weekly for a 3-week period. Each hybrid treatment was replicated eight times.

The mean damage rating of plants in the inoculated row increased over time while that in the adjacent rows largely remained the same (Figure 12).

#### 3.2.3. Destructive Sampling

**Figure 13:**
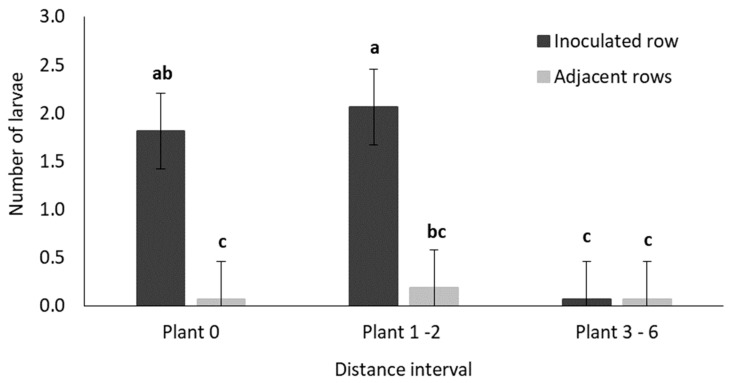
Mean (±SE) number of larvae recovered per distance interval (Figure 1) in the inoculated and adjacent rows of high-density (0.75 m inter-row; 0.18 maize intra-row spacing) and low-density (1.2 m inter-row; 0.42 m intra-row spacing) maize treatment plots in the field trial. Bars marked with different letters are significantly different (*p* < 0.05). The centermost maize plant in each treatment plot was inoculated with 30 MON810-resistant neonate *B. fusca* larvae (HAR18-R) at V4-growth, and the number of surviving larvae in each plant were recorded by destructive sampling of the treatment plots at the end of the trial. Each density treatment was replicated eight times.

The GLM analysis for average number of surviving larvae collected at the end of the field trial is reported in Table S4. The main effects (row and distance), as well as their interaction were significant (*p* < 0.05). Significantly higher numbers of larvae were recovered from plants in the first two distance intervals (up to two plants away from the inoculated plant) in the inoculated row (Figure 13). The mean number of larvae recovered per distance interval in the adjacent rows did not differ significantly (Figure 13).

## 4. Discussion

### 4.1. Importance of Laboratory, Semi-Field, and Field Experiments

This study highlighted the importance of observing insect pest behaviour in experimental setups that increase in scale and provide a close approximation of field conditions. In a different study, Visser et al. [44] reported that laboratory-based plant abandonment experiments indicated that the presence of Bt toxins in maize plants caused *B. fusca* larvae to balloon from plants at a higher rate compared to non-Bt maize plants. It was therefore expected that larvae would also migrate more extensively in Bt maize fields, which would lead to a greater number of plants showing symptoms of stem borer feeding damage. However, this was not the observation made in the field trials. Instead, more plants were damaged in non-Bt maize than Bt maize plots, highlighting the importance of testing hypotheses of insect pest behaviour in both small- and large-scale experiments. Han et al. [45] also highlighted this in their review paper on the importance of experimental scale and setup when measuring the effects of genetically engineered crops on the behaviour of both beneficial and pest insects.

### 4.2. B. fusca Larval Migration Behaviour

This study supports previous reports on migration behaviour of *B. fusca* larvae under field and semi-field conditions. Apart from the inherent pre-feeding migration behaviour described above and reported by Calatayud et al. [46], *B. fusca* larval migration was also reported to be affected by larval density [33,46,47]. The number of damaged plants and severity of damage only increased over the first three weeks after inoculation of larvae onto plants. By the 3rd week in both the greenhouse and field experiments, one to two plants adjacent to (and in the same row as) the natal plant exhibited increased damage symptoms. This suggests that larvae did not migrate beyond week 3, since the presence of a damaged plant reflected the position in the plot where larvae occurred at the end of the experiment. These data suggest that by 21 days after inoculation, the number of larvae per plant were low enough to preclude the need for further movement. Van den Berg et al. [48] reported similar results for *B. fusca* on grain sorghum, indicating that inoculation of a single plant resulted in damage to only approximately five plants within a row over a two-week period. Density dependent migration of lepidopteran larvae is supported by several studies [20,28,33,48,49,50,51,52,53,54,55,56,57,58].

### 4.3. B. fusca Larval Migration and Plant Density

The mean number of damaged plants, and the distance of damaged plants from the point of inoculation were always higher in the inoculated row than adjacent rows in both the greenhouse and field experiments. The mean damage rating was also consistently higher in the inoculated rows. Plants in adjacent rows displayed significantly higher damage ratings in high-density treatment plots indicating that larvae migrated across rows more easily in the high-density plots. At the end of the experiment, however, the overwhelming majority of larvae were recovered from the inoculated plant itself and only two plants away within the same row. A significantly lower number of larvae were recovered from adjacent rows in the greenhouse experiment, and virtually no larvae were obtained from adjacent rows in the field experiment. 

The effects of plant density on the migratory behaviour and survival of *B. fusca* larvae has also been investigated by other authors. A study conducted by Van Rensburg et al. [58] assessed the migration and survival of *B. fusca* in maize plots with differing inter- and intra-row spacings. They used inter-row widths of 0.9, 1.5, and 2.2 m, and intra-row spaces that allowed either 25, 35, 45 or 55 plants per 10 m row length. In that study, they reported that *B. fusca* larvae were highly mobile and were able to migrate approximately 3.6 m (across four inter-row spaces of 0.9 m) away from the natal plant. However, it is likely that the effect of natural infestation was not considered, since they reported an unprecedented larval survival rate of 72% after four weeks under field conditions. This calls into question the accuracy of the reported distance that the larvae migrated in the latter study. Despite this, Van Rensburg et al. [58] still observed marked differences in the incidence of damaged plants and numbers of surviving larvae in high-density and low-density plots. Other studies also found that the likelihood of successful larval migration is higher in denser plantings [59,60,61].

One of the earliest reports on the migration potential of *B. fusca* larvae also supports the findings of this study. Harris [62] described the migration of *B. fusca* larvae in smallholder maize and sorghum fields in Nigeria and reported a mean migration distance of 1.5 m within the row of initial infestation. The inter and intra-row spacing in these fields were however not reported which precludes direct comparison to the findings of this study. Yet, Harris [62] described the migration of *B. fusca* larvae by stating: “During the larval period, there is generally little migration away from the site of hatching. In insectary cages the maximum distance of dispersal from a known site of infestation was 2.0 m and, in the field, infestations which result from a discrete wave of oviposition during the early growing season, are virtually limited to the areas where eggs were laid.” More recently, Ndjomatchoua et al. [42] also indicated that the increased incidence of plants exhibiting whorl damage symptoms was due to interplant movement of larvae and that the clustered distribution of borer-damaged plants under field conditions was as a result of the initial random selection of a plant for oviposition by the female, followed by local short-range inter-plant movement of larvae.

### 4.4. B. fusca Larval Migration and Plant Growth Stage

The growth stage of plants at the time of infestation also had a significant effect on the spatial and temporal migration of larvae of *B. fusca* larvae. The low incidences of internode damage in this study attested to the unsuitability of older plants for colonization and development of young larvae. Few of the older plants were damaged and less severe tunnelling was observed in the late infested plants. During the early infestation, larvae that bored into stems preferred to do so in the lower part of the maize stem in all three hybrid treatments (data not shown). Plant structure and architecture play a major role in the survival and migration of larvae. Larger plants with less soft tissue and fewer suitable places for shelter limits the success of larvae to survive on plants which become infested at late growth stages, especially during the period just preceding tasselling [62,63,64,65,66]. 

Although all plants that were inoculated during the early vegetative stage suffered tunnel damage in the same internode zones, differences were observed in tunnel lengths for the three hybrid treatments (data not shown). The mean tunnel length recorded for non-Bt plants were significantly greater than that recorded in plants of MON89034. Internode damage data therefore indicate that plant growth stage at the time of infestation determines which plant parts suffer the greatest damage, whereas the presence of Bt toxin will determine the extent to which plants are damaged. This effect of Bt toxin on the degree of internode damage was also observed for two another maize stemborers, *O. nubilalis* and *Diatraea grandiosella* (Dyar) (Lepidoptera: Crambidae) [67,68,69,70]. 

### 4.5. B. fusca Larval Migration and Bt Toxins

Based on results obtained from the laboratory experiment, where larvae showed significant Bt avoidance behaviour (by ballooning off plants), it was expected that a greater number of plants would exhibit whorl damage symptoms in the Bt maize than non-Bt maize plots under greenhouse and field conditions. This hypothesis was also developed by Visser et al. [27], following the results from choice-test experiments that indicated that both Cry1Ab-resistant and susceptible larvae avoided feeding on MON810 and MON89034 leaf samples. However, the results of the greenhouse and field experiments did not support this hypothesis. These data suggest that larvae migrated more extensively and successfully in plots of non-Bt plants, and especially when compared to the MON89034 maize plants that the larval population is not resistant to. 

In the MON89034 plots in the greenhouse trial, the mean number of damaged plants, severity of damage, as well as the number of surviving larvae collected from plants other than the non-Bt inoculated plant were insignificant, regardless of plant growth stage at which inoculation was done. Additionally, no statistical difference was found between the incidence of damaged plants in the non-Bt and MON810 plots of both the early and late inoculated treatments, suggesting that the Cry1Ab-resistant larvae reacted to MON810 plants the same as they would to non-Bt plants. The assumption made from the laboratory experiment that larvae would migrate more between plants in Bt maize plantings was also contradicted by the results obtained in the field experiment, since the number of damaged plants in non-Bt maize plots were significantly higher than in MON810 plots. 

Erasmus et al. [71] conducted a similar study under field conditions over two seasons and compared the incidence of plants damaged by Cry1Ab-resistant *B. fusca* larvae in plots planted with seed mixtures of non-Bt and Bt maize. In their study, they used plots (7 × 5 m, with an inter-row spacing of 0.17 m and intra-row spacing of 0.90 m) of MON810 and MON89034 maize that contained different percentages of non-Bt maize plants distributed randomly within the plots of Bt maize, in order to simulate a seed-mixture scenarios. Fifty neonate *B. fusca* larvae of a Cry1Ab-resistant population were inoculated onto a non-Bt plant in the centre of each experimental plot, and the incidence of leaf feeding damage was recorded on a weekly basis for nine weeks. The 100% non-Bt control plots and 5% non-Bt treatment plots of their study closely resembles the non-Bt and MON810 field experimental plots of this study, and therefore warrant a direct comparison. 

Table 1 contains the results of incidence of damage per plot recorded by Erasmus et al. [71] three weeks after inoculation. In our study, the mean number of plants damaged in the non-Bt plots (9.75 plants) was lower than that recorded by Erasmus et al. [71] in non-Bt control plots, whereas the mean number of plants damaged in the MON810 plots (5.25 plants) was slightly higher than that observed in plots containing 5% non-Bt plants [71]. However, these differences are still comparable as they fall within the relative variation observed in both studies, especially when considering the natural infestation (5% and 3% in each of the two seasons) recorded in the study of Erasmus et al. [71]. These authors also inoculated more larvae per plant (50 larvae vs 30 larvae), and had a narrower inter-row spacing than that used in this study. 

Unfortunately, Erasmus et al. [71] did not assess larval damage to plants, nor did they report the distance between the inoculated plant and other plants where damage was recorded, which precludes comparison of results on those metrics. They did, however, monitor the larval survival each week in a separate plot of maize plants inoculated with 10 larvae each. They reported that, by 21 days after inoculation, a mean of 25% larvae in season 1 and 12% larvae in season 2 were recovered from each plant, indicating a very low larval survival rate. Since these larvae were inoculated onto non-Bt maize at very low densities, and every plant had the same number of larvae inoculated onto it, it could be assumed that larval migration was not the reason for the decrease in the larvae per plant, but rather a low larval survival rate. Their low survival rate was mirrored in this study: in the greenhouse experiments, the survival rate was 20.8% in early infestation, and 16.1% in late infestation treatment plots, whereas only 14.6% and 13.8% surviving larvae were recovered from high- and low-density treatment plots in the field (data not shown). High mortality of neonate lepidopteran larvae have essentially been established as the norm, since this has been observed in several studies [1,19,20,28,58,72,73,74]. Zalucki et al. [28] stated that commonly nearly 40% of eggs do not hatch due to egg parasitism and other biotic and abiotic factors, and that a further 50% of larvae perish during the first instar. The inoculated plants in the late infestation greenhouse plots had a mean number of 5 larvae recovered from the plants. This is greatly because the late infestation treatment was inoculated much later than the early infestation treatment, and that the conditions in the greenhouse improved the rate of survival to some extent, since larvae were protected from predators, parasitoids, and extreme abiotic conditions. 

The damage rating results recorded in the greenhouse echoed the damage frequency results. The mean damage rating in non-Bt plots did not differ statistically from MON810 plots but were significantly higher compared to the MON89034 plots up to two plants away from the inoculation point in the inoculated row. In the field, the damage rating was similar on both non-Bt and MON810 plants up until two plants from the point of inoculation. However, from three plants onwards, the damage rating was significantly higher in non-Bt plants. 

### 4.6. How Many Maize Plants Do the Larvae from a Single B. fusca Egg Batch Damage?

Results suggest that the larvae from an egg batch containing 30 eggs, laid on maize plants during the early vegetative stages would, over a three-week period, damage a mean total of 5 plants in MON810 and 10 plants in a non-Bt field, mostly within 1 m from the inoculated plant in the same row. Kaufmann [75] also reported that *B. fusca* neonate larvae immigrated immediately after eclosion and that an infestation level of 30 eggs per plant resulted in an eventual spread to three maize plants over time, similar to what is reported in this study. However, the degree of damage could range between severe destruction of the young leaves, to light (isolated holes (windowpanes) on some of the leaves), typically decreasing as the distance from the natal plant increases. Additionally, only approximately four larvae out of the original 30 would have survived up to three weeks after inoculation, and these larvae would likely be found no further than two plants away from the natal plant. In a study similar to the field trial, Pannuti et al. [19] evaluated the migration distance of *Striacosta albicosta* (Smith) and *S. frugiperda* (both Noctuid maize pests, similar to *B. fusca*) on non-Bt maize, and reported that most of the larvae were recovered from the inoculated plant or within the inoculated row. For *S. albicosta*, 75% of larvae were recovered within a radius of 1.7 m from the inoculated plant, and 92% or *S. frugiperda* larvae were recovered within 1.1 m radius from the inoculated plant. In these plots, the inter- and intra-row spacing were 0.76 m and 0.15 m, respectively, resulting in a high density of maize plants which would’ve facilitated the migration of the larvae. The results therefore indicate that the majority of *S. albicosta* and *S. frugiperda* larvae were unlikely to migrate across more than one row of maize and could be found within 11 and 7 maize plants in the same row from the inoculated plant, respectively. Pannuti et al. [19] also reported low survival rates for both species: an average survival percentage of 13.5% and 5.1% for *S. albicosta* and *S. frugiperda*, respectively.

Several studies highlighted the tendency of *B. fusca* larvae to inhabit plants singly by the end of the season as testament to their prolific migration tendencies [33,57,71]. However, we propose that the spread of larvae in a maize field can only partially be attributed to larval migration, whereas the oviposition behaviour of female moths in all probability contribute more to the ultimate wide distribution of the larvae observed in the field. The latter was also suggested by Ndjomatchoua et al. [42]. *Busseola fusca* females regularly lay 7–8 egg batches (varying in size, but with an average of around 33 eggs) on several plants throughout their lifetime [33,57,76], avoiding damaged maize plants and plants already containing an egg batch when selecting a suitable host [33,77]. The behaviour of the females also explains why *B. fusca* infestations are often aggregated in parts of a maize field [42,78]. With the progression of the season and the occurrence of a second generation, the number of moths laying eggs will have increased, which would lead to an increase in the numbers of plants with egg batches. Larval survival in the latter scenario will however be lower since plants will be at advanced growth stages, similar to what was observed in the greenhouse study.

## 5. Conclusions

This study allows for the better understanding of *B. fusca* larval migration behaviour on maize. This will inform decisions about possible resistance management strategies to implement for this pest in Africa. It has often been proposed that seed mixtures are not suitable for pests that are highly mobile during the larval stage, since the migration of larvae between plants could lead to a situation where larvae are exposed to sub-lethal doses of the Bt toxin, thereby accelerating the evolution of resistance. 

Although this study showed that *B. fusca* larvae do migrate between plants, the migration distance is short and survival is very low, even in pure stands of non-Bt maize. This study also highlights the fact that farming practices which limit the successful migration and survival of larvae could contribute to IRM. This study therefore suggests that future research should aim to model resistance evolution based on migration parameters identified in this study. This would allow development of IRM strategies that take into account the biology and behaviour of *B. fusca*, as well as challenges provided to IRM in small holder farming systems where the use of structured refuges is not practical.

## Figures and Tables

**Table 1:** Results from Erasmus et al., (2016) on larval migration of *Busseola fusca* in maize plots with different seed blend ratios.

Treatment		Number of Plants in Treatment Plot	Number of Plants Damaged at 21 Days	% of Plants Damaged at 21 Days
Season 1	100% Non-Bt	113	18.08	16
	5% Non-Bt, 95% MON810	123	8.61	7
	100% Non-Bt	180	28.80	16
	5% Non-Bt, 95% MON89034	174	17.40	10
Season 2	100% Non-Bt	125	20.00	16
	5% Non-Bt, 95% MON810	125	1.25	1
	100% Non-Bt	190	28.50	15
	5% Non-Bt, 95% MON89034	180	1.80	1

## Supplementary Materials

The following are available online at https://www.mdpi.com/2075-4450/11/1/16/s1, Table S1: Factorial analyses of variance of the number of damaged maize plants in glasshouse treatment blocks, the general linear model analysis of the percentage of plants damaged, and the general linear model analysis of the damage rating score of maize plants at various distance intervals from the point of inoculation (Figure 1). Table S2: The general linear model analysis of the number of surviving larvae in maize plants at various distance intervals from the point of inoculation (Figure 1) in glasshouse treatment blocks. Table S3: Factorial analyses of variance of the number of damaged maize plants in field treatment blocks, the general linear model analysis of the percentage of plants damaged, and the general linear model analysis of the damage rating score of maize plants at various distance intervals from the point of inoculation (Figure 1). Table S4: The general linear model analysis of the number of surviving larvae recovered from maize plants in the inoculated and adjacent rows at various distance intervals from the point of inoculation (Figure 1) in field trial treatment blocks.
