# Supplementary material for: Larval Migration Behaviour of Busseola fusca (Lepidoptera: Noctuidae) on Bt and Non-Bt Maize under Semi-Field and Field Conditions
Source: Insects. 2019 Dec 23;11(1):16. doi: 10.3390/insects11010016 (PMC7022793; doi:10.3390/insects11010016)
Supplement: Supplementary file 1 [file insects-11-00016-s001.pdf]

insects

MDPI

Table S1. Factorial analyses of variance of the number of damaged maize plants in glasshouse treatment plots, the general linear model analysis of the percentage of plants damaged, and the general linear model analysis of the damage rating score of maize plants at various distance intervals from the point of inoculation (Figure 1). Results provided are limited to the main factors and interactions that had a significant p-value.

	Source	SS	d.f.	MS	F-value	p-value
Early infestation	**Number of damaged maize plants**					
	Week	29.58	4	7.40	8.51	***
	Row	185.01	1	185.01	212.79	***
	Week * Row	10.78	4	2.70	3.10	*
	Error	78.25	90	0.87		
	**Percentage damaged maize plants**					
	Distance	16.88	2	8.44	108.77	***
	Row * Distance	11.81	2	5.90	76.08	***
	Error	23.20	299	0.08		
Late infestation	**Number of damaged maize plants**					
	Hybrid	3.36	2	1.68	5.50	**
	Row	40.50	1	40.50	132.55	***
	Hybrid * Row	2.58	2	1.29	4.23	*
	Error	16.50	54	0.31		
	**Percentage damaged maize plants**					
	Distance	10.51	2	5.25	106.16	***
	Row * Distance	10.63	2	5.31	107.38	***
	Error	8.86	179	0.05		
	**Damage rating score**					
Early infestation date	Week	89.19	4	22.30	12.62	***
	Hybrid	81.49	2	40.75	23.07	***
	Row	1077.56	1	1077.56	610.06	***
	Distance	922.21	2	461.11	261.05	***
	Week * Row	25.68	4	6.42	3.64	**
	Hybrid * Distance	56.89	4	14.22	8.05	***
	Row * Distance	1025.97	2	512.98	290.42	***
	Hybrid * Row * Distance	18.22	4	4.55	2.58	*
	Error	476.91	270	1.77		
Late infestation date	Week	33.31	2	16.65	32.26	***
	Row	152.79	1	152.79	295.99	***
	Distance	187.20	2	93.60	181.32	***
	Week * Row	29.21	2	14.60	28.29	***
	Week * Distance	35.50	4	8.88	17.20	***
	Hybrid * Distance	9.07	4	2.27	4.39	**
	Row * Distance	194.36	2	97.18	188.26	***
	Week * Row * Distance	37.31	4	9.33	18.07	***
	Error	83.63	162	0.52		

Significance indicated by * ($p<0.05$), ** ($p<0.01$), and ***($p<0.001$).

Table S2. The general linear model analysis of the number of surviving larvae in maize plants at various distance intervals from the point of inoculation (Figure 1) in glasshouse treatment plots. Results included were limited to the main factors and interactions that had a significant p-value.

Source	SS	d.f.	MS	F-value	p-value
Row	65.34	1	65.34	25.73	***
Distance	80.10	2	40.05	15.77	***
Infestation date * Distance	24.35	2	12.17	4.79	**
Row * Distance	68.18	2	34.09	13.43	***
Infestation date * Row * Distance	32.38	2	16.19	6.38	**
Error	274.25	108	2.54		

Significance indicated by * ($p<0.05$), ** ($p<0.01$), and ***($p<0.001$).

Table S3. Factorial analyses of variance of the number of damaged maize plants in field treatment plots, the general linear model analysis of the percentage of plants damaged, and the general linear model analysis of the damage rating score of maize plants at various distance intervals from the point of inoculation (Figure 1). Results included were limited to the main factors and interactions that had a significant p-value.

Source	SS	d.f.	MS	F-value	p-value
Number of damaged maize plants					
Plant density	22.04	1	22.04	10.24	**
Week	22.27	2	11.14	5.17	**
Hybrid	28.17	1	28.17	13.08	***
Row	108.38	1	108.38	50.34	***
Error	155.00	72	2.15		
Percentage of damaged maize plants					
Distance	11.67	2	5.83	74.43	***
Row * Distance	8.42	2	4.21	53.77	***
Error	19.83	253	0.08		
Damage rating score					
Plant density	25.39	1	25.39	11.72	***
Week	265.57	2	132.79	61.28	***
Hybrid	43.54	1	43.54	20.09	***
Row	598.05	1	598.05	275.99	***
Distance	432.67	2	216.34	99.83	***
Plant density * Row	11.35	1	11.35	5.24	*
Week * Row	84.98	2	42.49	19.61	***
Week * Distance	64.82	4	16.21	7.48	***
Hybrid * Distance	20.11	2	10.05	4.64	**
Row * Distance	521.91	2	260.95	120.42	***
Week * Row * Distance	53.41	4	13.35	6.16	***
Error	468.06	216	2.17		

Significance indicated by * (p<0.05), ** (p<0.01), and ***(p<0.001).

Table S4. The general linear model analysis of the number of surviving larvae recovered from maize plants in the inoculated and adjacent rows at various distance intervals from the point of inoculation (Figure 1) in field trial treatment plots. Results included were limited to the main factors and interactions that had a significant p-value.

Source	SS	d.f.	MS	F-value	p-value
Row	35.04	1	35.04	14.09	***
Distance	20.59	2	10.29	4.14	*
Row * Distance	17.58	2	8.79	3.54	*
Error	179	72	2.49		

Significance indicated by * (p<0.05), ** (p<0.01), and ***(p<0.001).
